# Application of CareDose 4D combined with Karl 3D technology in the low dose computed tomography for the follow-up of COVID-19

**DOI:** 10.1186/s12880-020-00456-5

**Published:** 2020-05-24

**Authors:** Jiawei Li, Xiao Wang, Xiaolu Huang, Fangxing Chen, Xuesong Zhang, Ying Liu, Guangzuo Luo, Xunhua Xu

**Affiliations:** 1 Department of Radiology, China Resources & WISCO General Hospital, Wuhan, 430080 China; 2 Department of Biochemistry and Molecular Biology, China Medical University, Shenyang, 110122 China; 3 Institute of Translational Medicine, China Medical University, Shenyang, 110122 China

**Keywords:** COVID-19, Radiation dose, CareDose 4D, Image quality

## Abstract

**Background:**

Coronavirus disease 2019 (COVID-19) is a highly infectious disease caused by the new coronavirus. Previous studies have shown that the chest CT examination plays an important role in the diagnosis and monitoring of COVID-19. However, some patients with COVID-19 had low white blood cell counts and reduced lymphocyte ratios. Multiple CT examinations may cause radiation damages as well as increase the apoptosis of peripheral blood lymphocytes. A new low-dose CT method should be developed because the regular CT may aggravate the disease.

**Method:**

Sixty cases were randomly divided into the study group (*n* = 30) and control group (n = 30). The lung window was reconstructed by Karl 3D iterative technique in the study group. The image quality was subjectively evaluated by two senior chest group diagnostic physicians using a 5-point double-blind method. The value of CT measurement and its standard deviation (SD) was used as an objective evaluation criteria. The volume of CT dose index (CTDI_vol_), dose length product (DLP) and effective dose (ED) from the two groups were compared and analyzed statistically.

**Result:**

There was no significant difference in the occurrence rates of ground glass opacities, consolidation, crazy-paving pattern, fiber cable shadow and axial interstitial thickening between the study group and control group (*p* > 0.05). In addition, no significant difference was found for the subjective score of overall image quality and image noise level (SD) between the two groups (*p* > 0.05). However, significant differences was found in CTDI_vol_, DLP, and ED between the study group and the control group (*p < 0.05*). The effective dose of the study group was reduced by 76% compared to the control group.

**Conclusion:**

CareDose 4D low-dose scanning combined with Karl 3D iterative reconstruction technology can not only greatly reduce the radiation dose, but also provide images that meet the diagnostic criteria of COVID-19, which can be used as a routine method for the follow-up of COVID-19 patients.

## Background

Coronavirus disease 2019 (COVID-19) is a highly infectious disease caused by the new coronavirus [1, 2]. Timely and effective intervention can improve the success rate of treatment of critically ill patients [3]. We believe imaging examination is the main method for clinicians to understand the progress of the disease. Previous studies have indicated that the chest CT examination plays a important role in the diagnosis and monitoring of COVID-19. However, some patients with COVID-19 had low white blood cell counts and reduced lymphocyte ratios [4]. Multiple CT examinations may cause radiation damages as well as increase the apoptosis of peripheral blood lymphocytes [5]. In this study, we have explored the application of low-dose chest CT scans in the follow-up of patients with COVID-19 using Care Dose 4D combined with Karl’s 3D iterative reconstruction technology compared with conventional-dose chest CT.

## Methods

### General materials

Fifty-six patients with common COVID-19 diagnosed in our hospital from February 17, 2020 to March 4, 2020 were selected, including 29 males and 27 females aged 32 to 86 years with an average age of (61.9 ± 13.7) years. CT scan was performed within 2–3 weeks after diagnosis. All cases with body mass index (BMI) in the range of 18.5 ~ 24.9 kg / m^2^ were randomly divided into a study group and a control group with 30 cases in each group (4 patients from the two groups had two CT follow-ups performed by the same patient at different times). There were 17 males and 13 females in the study group, aged 38 to 81 years with an average age of (61.6 ± 12.6) years. There were 13 males and 17 females in the control group, aged 32 to 86 years, with an average age of (62.2 ± 15.0) years. Inclusion criteria: ① at least one positive test of the new coronavirus nucleic acid; ② complete clinical data and CT thin-layer images of the chest. Exclusion criteria: ① patients with underlying lung diseases, such as lung tumors, tuberculosis, and other infections in the lungs; ② patients could not cooperate for some reason, which led to blurred CT images.

### Inspection method

The equipment used for lung scan is a 40-row multi-slice spiral CT (uCT530, United Imaging, Inc., China). For CT inspection, the patient was in a supine position, with both hands raised above the head. The scans were performed from the apex of both lungs to the diaphragm on both sides when the breath of the patients was hold at the end of deep inhale. CareDose 4D technique and filtered back projection(FBP) algorithm image reconstruction were used in both groups. The fixed tube current of the two groups was 120 kV. The reference tube currents of the study group and the control group were 30mAs and 130mAs, respectively. The scan matrix was 512 × 512, with 5 mm of layer thickness, 5 mm of layer spacing and 1.0725 of pitch. The image reconstruction layer thickness was 1 mm and the layer spacing was 0.8 mm. The lung window of the control group was reconstructed with routine reconstruction method, while the study group was reconstructed using Karl 3D level 3 iterative technology. The KARL Iterative Reconstruction technique is designed to perform both in the projection (raw data) space and the image space for uCT family systems. Starting in projection space, KARL detects signals that are likely to contribute to image artifacts. Once the signals are selected, their noise level will be estimated based on photon statistics. Then the projection data will be iteratively processed by penalizing the selected noise data according to the noise model, and artifacts would be significantly suppressed while edge information is still preserved.

### Image evaluation

The personal information, scan parameters and dose of the patients were hidden in the PACS system. The images were evaluated by two senior chest diagnosticians using double-blind method at fixed window position and width. The parameters for lung window are − 600 HU of position and 1200 HU of width. The parameters for mediastinal window are 40 HU of position and 350 HU of width. The image quality of ground glass opacities (GGO) and crazy-paving pattern were scored subjectively according to the 5-point scale evaluation system.

#### Subjective evaluation

Five-point scale system was used to evaluate the images (Table 1). A score of 3 or more is considered to meet diagnostic requirements. In case of the two physicians do not agree on the score, they will reach an agreement through consultation.

**Table 1:** Subjective evaluation standard of 5-point system

Score	GGO	Crazy-paving pattern
Five points	clear	clearly visible
Four points	clear	visible
Three points	visible	unclear
Two points	visible	blurred
One point	non-visible	unclear

#### Objective evaluation

The CT values were measured and standard deviation (SD) was calculated for an area of about 1 cm^2^ area of interest (ROI) in the central area of the descending aorta at the tracheal carina layer. The volume of CT dose (CTDIvol) and the dose length product (DLP) were automatically given by the recording system, which were used to calculate the effective dose (ED), ED = DLP × k (chest k = 0.014).

#### Statistical analysis

The SPSS 17.0 statistical software is used for statistical analysis. All quantitative data are expressed as x ± s, and qualitative data are expressed as percentages. The independent sample *t*-test was used to measure the differences in subjective quality scores, CT values of descending aorta, SD of image noise, CTDIvol, DLP and ED of the images between the control group and the study group. The incidence rates of GGO, consolidation, crazy-paving pattern, fiber cable shadow and central axis interstitial thickening between the two groups were compared using the x^2^ test. *p* < 0.05 was considered statistically significant.

## Results

### Comparison of the CT signs

**Fig. 1:**
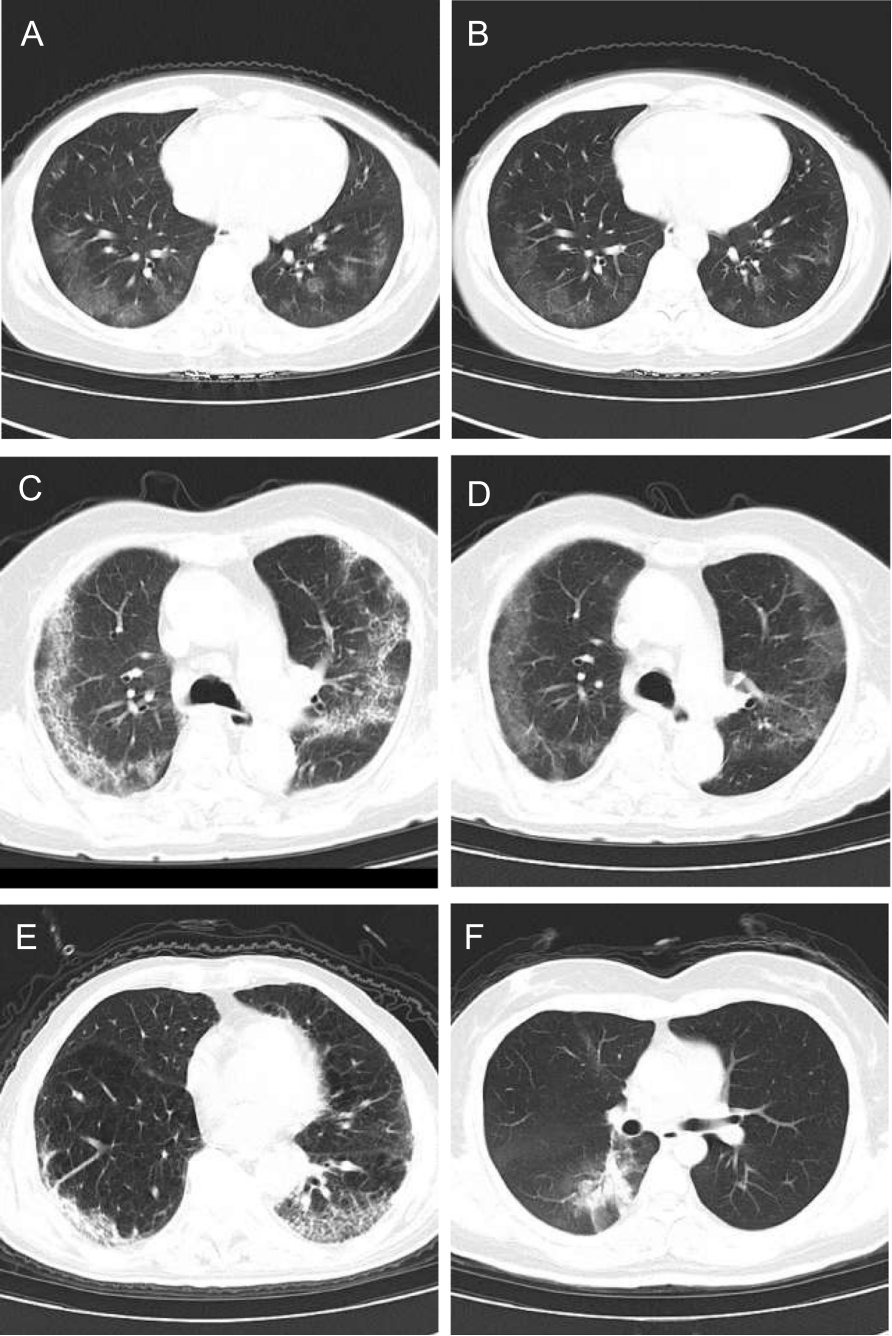
CT images using CareDose 4D combined with Karl 3D technology in the low dose for the follow-up of COVID-19. **(A-B)** CT images of a 43-year old female. Follow-up with low-dose CT (Fig. A) showed that the lower lobe of the lungs is scattered with thin GGO and the edges are blurred. Tube voltage is 12 0 kV and tube current is 29 mA. After 5 days, follow-up with conventional dose CT (Fig. B), showed slightly absorbed sub-pleural GGO compared to Fig. A. Tube voltage is 120 kV and tube current is 20 mA. **(C-D)** CT images of a 81-year old female. Follow-up with conventional dose CT (tube voltage of 120KV and tube current of 131 mA) curved grid-like shadows can be seen under the pleura of both lungs, showing crazy-paving pattern (Fig. C), followed by low-dose CT (tube voltage of 20 kV and tube current of 31 mA) follow-up 2 weeks (Fig. D), The symptoms are not obvious, and the arc GGO changes. **(E)** CT image of a 64-year old male. Low-dose CT follow-up of clearly showed that the lower lobe subpleural crazy-paving pattern and the right lower lung was partially consolidated. **(F)** CT image of a 38-year old female. Tube voltage 120 kV, tube current 43mAs. Follow-up with low-dose CT showed flaky consolidation in lower lobe of right lung. Dissipative GGO was seen in the surrounding area. Tube voltage is 120 kV and tube current is 33 mA

The number of cases with multiple lesions in the study group and control group are 26 and 24, respectively. There were 4 cases with diffusely distributed CT sign in the study group and 6 case in the control group. For cases with mainly subpleural distribution, the number is 28 and 25 for the study group and control group, respectively. There were 2 cases in the study group and 5 cases in the control group were distributed along the bronchial vascular bundle. The main signs of the two groups (Fig. 1 A ~ F) include GGO, crazy-paving pattern, consolidation and fiber cable shadow. A few patients may have axial interstitial thickening. There were no statistical differences in the occurrence rate of the above signs between the two groups (*p* > 0.05, Table 2).

**Table 2:** Comparison of the incidence of CT signs (%)

CT signs	GGO	Consolidation	Crazy-paving pattern	Fibrous cord shadow	Axial interstitial thickening
Control group	26 (86.7)	23 (76.7)	21 (70)	14 (46.7)	3 (10)
Study group	24 (80)	21 (70)	20 (66.7)	14 (46.7)	4 (13.3)
*x*^*2*^ value	0.48	0.34	0.077	0.00	0.16
*p* value	0.49	0.56	0.78	1.00	0.69

### Comparison of image quality

The subjective overall image quality score of the study group was 3.97 ± 0.81, which was not significantly different from that (4.27 ± 0.78) of the control group (*p* > 0.05). We also did not found significant difference in CT value and SD of descending aorta between the control group and the study group (*p* > 0.05, Table 3).

**Table 3:** Comparison of subjective score, CT value, and SD value of descending aorta

	Subjective score	Descending aortic CT value	SD
Control group	4.27 ± 0.78	36.03 ± 5.78	7.27 ± 0.42
Study group	3.97 ± 0.81	38.06 ± 5.73	7.44 ± 0.29
*t* value	1.46	−1.37	−1.86
*p* value	0.15	0.17	0.07

**Note:** Subjective score, descending aortic CT value, and SD value are expressed as x ± s

### Comparison of radiation dose

Although there was no significant difference in CT signs and image quality between the study group and control group, the differences in CTDIvol, DLP, and ED between the two groups were statistically significant (t = 31.14, 27.73, 27.73, *p* < 0.01, Table 4). The effective dose in the study group was reduced by 76% compared to the control group.

**Table 4:** Comparison of radiation dose(x ± s)

	CTDI_vol_ (mGy)	DLP (mGy.cm)	ED (mSv)
Control group	11.21 ± 1.50	360.50 ± 52.99	5.05 ± 0.74
Research group	2.53 ± 0.27	87.25 ± 10.21	1.22 ± 0.14
*t* value	*31.14*	*27.73*	*27.73*
*p* value	0.00	0.00	0.00

Note: CTDIvol, DLP and ED are expressed as x ± s

## Discussion

The chest CT scan can be used for the follow-up of COVID-19 to guide clinical management [6]. COVID-19 has made rapid progress, and its imaging signs have changed greatly in different periods. Multiple chest CT examinations can help monitor the progression change of COVID-19 disease, which has important value in the follow-up of COVID-19 [7]. However, multiple CT follow-ups will increase medical exposure, which may exaggerate disease condition.

How to reduce the radiation dose under the premise of meeting the needs of the imaging diagnosis has been studied intensively in recent years. Among them, reducing the tube current is one of the main methods to reduce the radiation dose. Studies show that lowering the tube current can effectively reduce the CT radiation dose [8]. Chinese Medical Association experts have recommended 100 ~ 120KV and less than 30mAs as a new generation iterative algorithm of low-dose screening scheme CT [9]. Hu et al. have screened ground glass nodules in the lungs using the reference tube current of 30 mAs. They used CareDose 4D technology to automatically adjust the tube current according to the change of the thickness of the human body to obtain similar image quality for each layer [10]. Although low-dose scanning has been studied intensively, no personalized scanning method specifically for GGO and crazy-paving pattern has been reported. In this study, we have applied a CareDose 4D scanning method combined Karl 3D iterative reconstruction technology with a fixed 120 kV and reference tube current of 30mAs for the follow-up of COVID-19 patients. We have used Karl 3D iterative reconstruction technology in the study group, but not in the control group. If the control group of the lung window use karl 3D iterative reconstruction, the images will be too smooth, and the contrast will be reduced. Karl 3D is an iterative reconstruction technology of United Imaging Healthcare (UIH), which found that image quality is limited by conventional filtered back projection (FBP) imaging reconstruction technique. By FBP, noise in reconstruction images obviously increases as radiation dose decreases, which impacts diagnostic confidence. In order to effectively reduce radiation dose while ensuring the accuracy of diagnosis, Karl 3D iterative reconstruction technology has been developed by UIH, which can greatly reduce the image noise caused by low-dose scanning and effectively improve the image quality [11]. We found that there was no statistically significant difference in the objective scores of overall image quality between the study group and control group. However, significant differences were found in CTDIvol, DLP and ED radiation dose indexes between the two groups. The effective dose in the study group (1.22 ± 0.14 mSv),which was reduced by about 76%, was much lower than that of the control group (5.05 ± 0.74 mSv).

Previous studies have shown that the CT image results showed that small patchy GGOs are usually found to be localized at the pleura, unilateral or lower lung lobe at early stage of COVID-19 [12]. As the disease progresses, GGO will increase and some consolidations and grid-like changes may also occur. Two weeks later, the lesions are gradually absorbed. The density, scope and number of lesions is reduced. Only a little thin GGO and fiber strand shadows remain. During the early GGO progress, the density increases and the interlobular septal thickening forms a grid-like shadow, which is the crazy-paving pattern [13, 14]. We used GGO and crazy-paving pattern as the image evaluation criteria for two main reasons. One is that these two signs are important CT signs of COVID-19, and the occurrence rate is high. The second is that the GGO contrast is low and close to normal lung tissue. It is difficult to observe the lesion clearly when the image noise is increased [15]. The main CT signs of COVID-19 include GGO, consolidation, crazy-paving pattern, and fiber strands, etc. But axial interstitial thickening is rare. It may be related to the fact that the lesion is easy to involve the peripulmonary interstitium and less axial interstitial tissue. We found that the incidence of the above signs was similar and there was no statistical difference between the two groups. In our study, the subjective scores of the image quality of the two groups were ≥ 3, with no statistical difference. Also, there was no significant difference in the SD value between the two groups. Although the image quality of the study group was slightly lower than that of the control group, it meets the imaging diagnostic requirements. The high incidence of multiple signs in this study indicates that the disease progresses rapidly, and signs often coexist in multiple periods.

We have noticed that there are some limits in our study. For example, the small sample size may lead to biased statistical analysis results. Also, only a set of low-dose scanning protocols has been established. Further experiments are required for a multi-parameter controlled study, which will make our conclusion more solid.

## Conclusion

Our finding suggests that it is feasible to follow up patients with COVID-19 using the method of CareDose 4D combined with Karl 3D technology in the low dose computerized tomography. Under the condition of ensuring the image quality, the radiation dose is reduced to a great extent.

## Data Availability

The full data sets used and analyzed during the current study are available on reasonable request from the corresponding authors.

## Abbreviations

CT: Computed tomography
COVID: Coronavirus disease
SD: Standard deviation
CTDIvol: CT dose index
DLP: Dose length product
ED: Effective dose
